# Cutaneous Branch of the Spinal Accessory Nerve: Case Report With Potential Relevance to Occipital Neuralgia

**DOI:** 10.7759/cureus.17666

**Published:** 2021-09-02

**Authors:** Aditi Patel, Caroline Watson, Łukasz Olewnik, Joe Iwanaga, R. Shane Tubbs

**Affiliations:** 1 Anatomy, Ross University School of Medicine, Two Mile Hill, BRB; 2 Department of Ophthalmology, Tulane University School of Medicine, New Orleans, USA; 3 Department of Anatomical Dissection and Donation, Medical University of Lodz, Lodz, POL; 4 Department of Neurosurgery, Tulane University School of Medicine, New Orleans, USA; 5 Department of Structural & Cellular Biology, Tulane University School of Medicine, New Orleans, USA; 6 Neurosurgery, Ochsner Neuroscience Institute, Ochsner Health System, New Orleans, USA

**Keywords:** surgery, occiput, skin, innervation, posterior cervical triangle, anatomy

## Abstract

We describe a case in which a cutaneous branch was found arising from the spinal accessory nerve, a nerve typically characterized as a purely motor nerve. Although reported anatomical variations of the lesser occipital and spinal accessory nerves are uncommon, rare variants have been reported. Such anatomy might result in unexpected patient presentations or rare complications following spinal accessory nerve injury.

## Introduction

The spinal accessory nerve (SAN) is the 11th cranial nerve and is derived from motor neurons located in the upper cervical spinal cord and medulla oblongata and innervates the sternocleidomastoid and trapezius muscles. The nerve leaves the skull base via the jugular foramen and usually, travels anterior to the internal jugular vein to then enter the posterior surface of the sternocleidomastoid muscle. This from approximately the junction of the upper fourth and lower three-fourths of the sternocleidomastoid, the nerve leaves the muscle’s posterior border to descend superficially in the posterior cervical triangle to enter the trapezius muscle [1,2]. It is not described as typically containing any sensory fibers, specifically, cutaneous branches [1-3]. The sternocleidomastoid laterally flexes and rotates the neck and the trapezius elevates, retracts, and rotates the scapula.

The lesser occipital nerve (LON) is a cutaneous nerve arising from the cervical plexus and innervates the posterior auricular area, as well as portions of the lateral scalp over the occiput. The nerve usually arises from C2 or C2/C3 spinal nerve levels and ascends along the posterior border of the sternocleidomastoid muscle toward the cutaneous regions it supplies. 

Although reported anatomical variations of the LON and SAN are uncommon, rare variants have been reported. For the LON, reported variations include duplication of its branches and for the SAN, a duplicated nerve, varying connections with the C1 spinal nerve and its ganglion, and connections with the nearby facial nerve [3]. Here, we describe a case in which a cutaneous branch arose from the SAN. To our knowledge, such a variant of the SAN previously has not been reported [4]. As such an anatomical variant could have clinical implications such as iatrogenic injury during surgical procedures over the occiput or posterior cervical triangle, knowledge of these uncommon anatomical variations are important to those treating patients with nearby pathology.

## Case presentation

**Figure 1:**
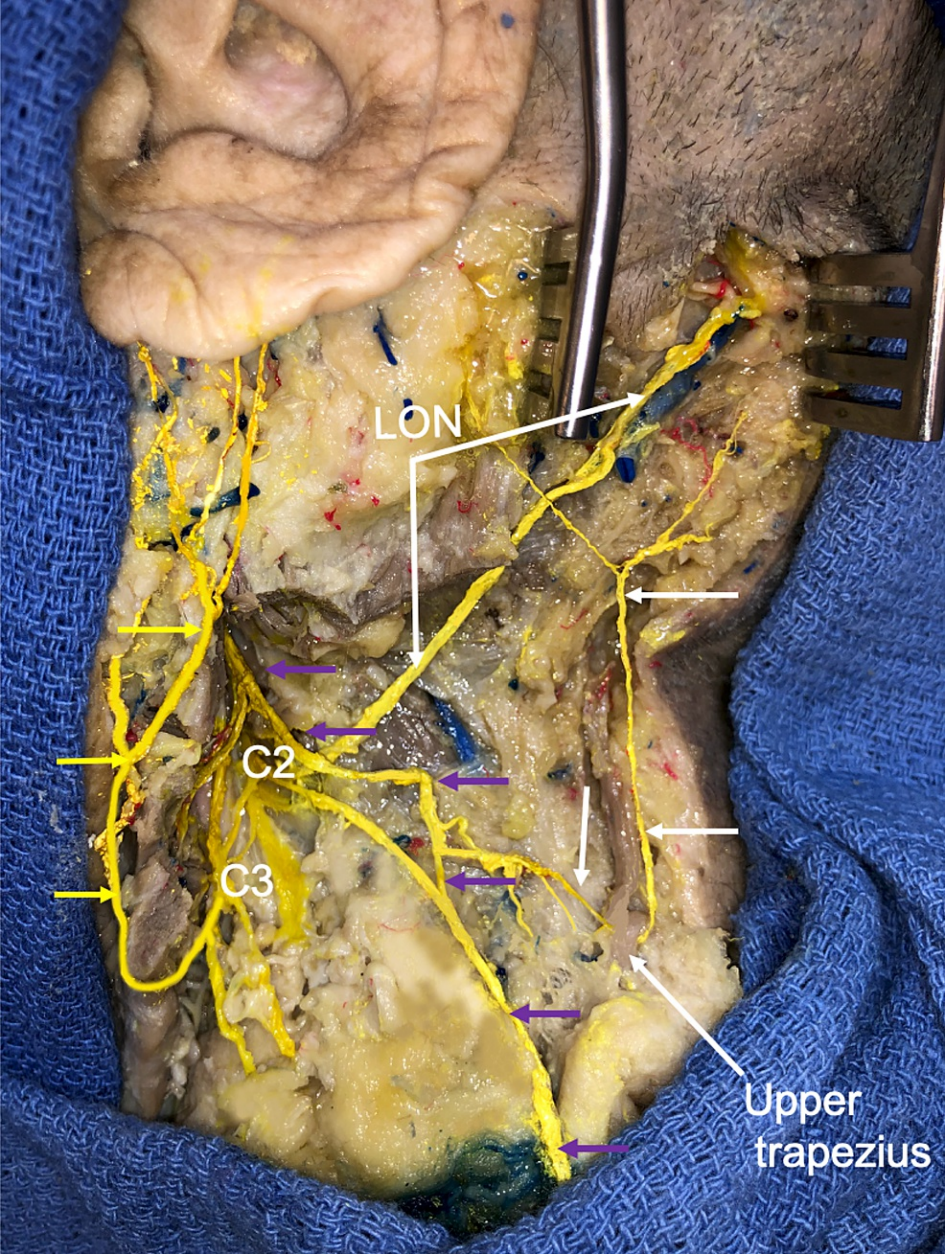
Cadaveric dissection of the left posterior cervical triangle and occiput. Note the spinal accessory nerve (SAN) (purple arrows) and lesser occipital nerve (LON). Also observe the cutaneous branch (white arrows) of the SAN that arises from an upper branch to the trapezius muscle and ascends to the mastoid and occipital regions. The more anteriorly placed great auricular nerve is shown at the yellow arrows.

**Figure 2:**
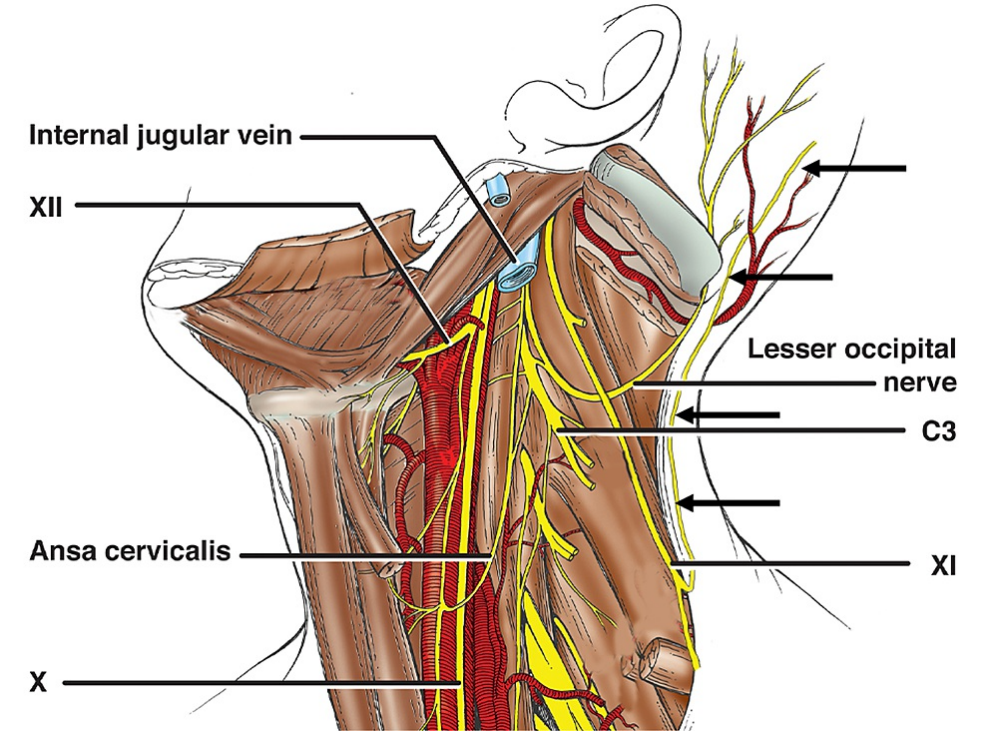
Schematic drawing of the case presented herein. The spinal accessory nerve (SAN) is shown descending superficial to the levator scapulae muscle and its upper branch to the trapezius recurs to travel to the skin over the mastoid and occipital regions as a cutaneous branch (arrows).

During the routine dissection of the left posterior cervical triangle in an adult male cadaver, an unusual branch of the SAN was identified. The cadaver was 83 years old at death. Following the removal of the skin and overlying fasciae from the posterior cervical triangle and occipital region, the SAN was traced from the skull base to its periphery. The sternocleidomastoid muscle was removed and the upper cervical nerve roots dissected. The nerves of this region were colored for clarity. In the posterior cervical triangle, the SAN was noted to give rise to a cutaneous branch measuring 0.9 mm in diameter and 7.1 cm in length that terminated as two terminal branches (Figures 1, 2).

The branch ascended parallel to the lateral border of the upper fibers of the trapezius muscle and with its lateral branch supplied the skin overlying the mastoid region and with its medial branch supplied the skin over the lower occiput. The later branch traveled more or less parallel with the LON. This cutaneous branch of the SAN was superficial to and slightly medial to the normally branching LON and with this nerve, formed a neural decussation pattern (see Figure 1). With continued dissection, the SAN branch to the sternocleidomastoid was found to pierce the muscle and join the C2 ventral ramus. The C2 ventral ramus had a superior branch that became the LON and an inferior branch that joined the continuation of the SAN to the middle and lower parts of the trapezius muscle. This inferior branch also joined a smaller upper branch to the trapezius and these two fibers traveled laterally to give off descending subcutaneous branches that then pierced the upper trapezius fibers to ascend with the muscle. The LON traveled deep to the proximal part of the SAN and had a typical distal branching pattern over the occipital region. No additional neurovascular variations were noted in the left neck and no similar finding of the right SAN was found. There were no signs of previous trauma or surgery to the areas dissected. 

The authors sincerely thank those who donated their bodies to science so that anatomical research could be performed. Results from such research can potentially increase mankind’s overall knowledge that can then improve patient care. Therefore, these donors and their families deserve our highest gratitude [5].

## Discussion

The SAN contains two components: cranial and spinal. The cranial portion arises from the medulla oblongata and the spinal portion arises from neurons located in the anterior horn of the upper spinal cord, specifically C1 to C5. The spinal rootlets of the SAN travel upward through the spinal canal, through the foramen magnum, to join the cranial root. This union exits the skull laterally through the jugular foramen. Here, the SAN forms anastomoses with the vagus nerve [3]. This anastomosis has subjected the cranial portion of the SAN to controversy with some proposing that the cranial portion should be classified as part of the vagus nerve. After its passage through the jugular foramen, the spinal portion of the SAN then exits the skull, descending along the internal jugular vein, eventually entering the upper portion of the sternocleidomastoid muscle. It exits the muscle from the posterior surface, passing through the posterior cervical triangle. It then passes beneath the anterior border of the trapezius muscle where it enters the muscle. 

The path of the SAN has some known variants. For example, there are four documented pathways of the connection between the SAN and C1 rootlets and when present, C1’s dorsal root ganglion [4]. Type 1 and 2 variants do not have any connections with the posterior C1 nerve root, whereas types 3 and 4 have connections between the SAN and posterior C1 roots via varying routes [4]. Additionally, during its exit from the skull, the SAN descends along the internal jugular vein. It typically follows the vein laterally, though it has been noted to do so medially, as well as split along the medial and lateral sides, then rejoining later [4]. There have also been cases where the SAN passes through the internal jugular vein [6,7]. There are also variations in the path the SAN takes to exit the sternocleidomastoid muscle, such as a variation in which the SAN divides and forms a direct connection with the facial nerve and both nerves then innervate the sternocleidomastoid muscle [8]. The SAN elongates during the second and third months of embryological development [1]. Therefore, it is most likely during this time period that the anatomical variant as seen in our case report would take place while early relations between muscle masses and regional neurovascular structures.

The SAN is the only nerve providing motor innervation to the sternocleidomastoid muscle. It is the primary source of motor innervation to the trapezius muscle, although some motor innervation is also provided by the cervical plexus with varying modes of innervation via C2, C3 and/or C4 spinal nerves [9]. Clinically, this bears significant relevance in the case of nerve injury due to the limited motor innervation. 

The LON arcs around the SAN and ascends along the posterior border of the sternocleidomastoid muscle. The nerve then branches into auricular, occipital and mastoid branches [10]. In some cases, the LON passes through the sternocleidomastoid muscle. In other cases, it has even been noted to cross over the levator scapulae [11]. Variations have also been noted in the branch patterns of the nerve. For instance, on occasion, the auricular branch will arise from the greater occipital nerve rather than the LON. There are two known pathways of the LON. Type 1 nerves will stay along the posterior border of the sternocleidomastoid muscle until its insertion, after which the nerve begins to ascend, but type 2 nerves will ascend without following the sternocleidomastoid muscle border [11]. There have also been cases in which there was duplication of the nerve, or even a case in which there were three distinct LONs observed [12]. In some situations, the LON is small and supplies only a small area of skin on the neck. In these cases, the greater occipital nerve supplies the other areas which would typically be supplied by the LON. Notably, the LON has also been seen to arise directly from the SAN and communicate with the posterior auricular branch of the facial nerve [8]. One instance described a variation of the LON in which it provided communicating branches to the SAN [13].

In this case, a normal LON was observed with an additional branch superficial to it that arose from the SAN. Previous cases have been described in which the LON itself arises from the SAN [5], or when there are communicating branches between the two nerves [13]. However, the case reported herein is unique in that it had a normal LON with an additional cutaneous branch arising from the SAN overlying it. Again, while the SAN has primarily been characterized as a motor nerve, some studies suggest that it might also carry sensory or nociceptive information [14].

The most significant clinical relevance of the LON is its involvement in occipital neuralgia. The many variations in nerve branching patterns and origin make it difficult to efficiently treat the condition [15]. Moreover, if a patient were to present with symptoms of occipital neuralgia, a case such as reported here would be confounding to the diagnosis. The surgical significance of the SAN is primarily in its iatrogenic injury. For example, surgeons performing lymph node biopsy of the posterior cervical triangle are at risk of injuring the SAN. Additionally, the SAN is often used for neurotization procedures of, for example, branches of the brachial plexus such as the suprascapular nerve [2,7]. 

## Conclusions

To our knowledge, no previous reports have documented a cutaneous branch of the SAN in the English literature. Future studies aimed at any cutaneous sensory deficits following SAN injury would help clarify this variant anatomy.

## References

[1] (2021). Gray's Anatomy 42nd edition. https://evolve.elsevier.com/cs/product/9780702077050?role=student.

[2] Ouaknine G, Nathan H (1973). Anastomotic connections between the eleventh nerve and the posterior root of the first cervical nerve in humans. J Neurosurg.

[3] Johal J, Iwanaga J, Tubbs K, Loukas M, Oskouian RJ, Tubbs RS (2019). The accessory nerve: a comprehensive review of its anatomy, development, variations, landmarks and clinical considerations. Anat Rec (Hoboken).

[4] Tubbs RS, Ajayi OO, Fries FN, Spinner RJ, Oskouian RJ (2017). Variations of the accessory nerve: anatomical study including previously undocumented findings-expanding our misunderstanding of this nerve. Br J Neurosurg.

[5] Iwanaga J, Singh V, Ohtsuka A (2021). Acknowledging the use of human cadaveric tissues in research papers: recommendations from anatomical journal editors. Clin Anat.

[6] Saman M, Etebari P, Pakdaman MN, Urken ML (2011). Anatomic relationship between the spinal accessory nerve and the jugular vein: a cadaveric study. Surg Radiol Anat.

[7] Hashimoto Y, Otsuki N, Morimoto K, Saito M, Nibu K (2012). Four cases of spinal accessory nerve passing through the fenestrated internal jugular vein. Surg Radiol Anat.

[8] Tubbs RS, Shoja MM, Loukas M, Lancaster J, Mortazavi MM, Hattab EM, Cohen-Gadol AA (2011). Study of the cervical plexus innervation of the trapezius muscle. J Neurosurg Spine.

[9] Yu M, Wang SM (2021). Anatomy, Head and Neck, Occipital Nerves. https://pubmed.ncbi.nlm.nih.gov/31194370/.

[10] Pantaloni M, Sullivan P (2000). Relevance of the lesser occipital nerve in facial rejuvenation surgery. Plast Reconstr Surg.

[11] Tubbs RS, Fries FN, Kulwin C, Mortazavi MM, Loukas M, Cohen-Gadol AA (2016). Modified skin incision for avoiding the lesser occipital nerve and occipital artery during retrosigmoid craniotomy: potential applications for enhancing operative working distance and angles while minimizing the risk of postoperative neuralgias and intraoperative hemorrhage. J Clin Neurosci.

[12] Madhavi C, Holla SJ (2004). Triplication of the lesser occipital nerve. Clin Anat.

[13] Ravindra S S, Sirasanagandla SR, Nayak SB, Rao Kg M, Patil J (2014). An anatomical variation of the lesser occipital nerve in the "carefree part" of the posterior triangle. J Clin Diagn Res.

[14] Bremner-Smith AT, Unwin AJ, Williams WW (1999). Sensory pathways in the spinal accessory nerve. J Bone Joint Surg Br.

[15] Dash KS, Janis JE, Guyuron B (2005). The lesser and third occipital nerves and migraine headaches. Plast Reconstr Surg.

